# The QuickSee autorefractor reliably measures the accommodative response in children compared with the Shin‐Nippon autorefractor

**DOI:** 10.1111/opo.13557

**Published:** 2025-07-19

**Authors:** Jane M. Fulton, Rebecca E. Leighton, Tuyishime Didier Fidele Uwacu, Bruce Moore, Julie‐Anne Little

**Affiliations:** ^1^ Centre for Optometry and Vision Science, School of Biomedical Sciences Ulster University Coleraine UK; ^2^ New England College of Optometry Boston Massachusetts USA

**Keywords:** accommodation, children, hyperopia, QuickSee, refractive error, vision screening

## Abstract

**Introduction:**

This study evaluated the QuickSee autorefractor for measurement of the accommodative response in a cohort of children without significant ametropia, and compared the findings to those determined using the gold‐standard Shin‐Nippon device.

**Methods:**

Children aged 5–7 years were recruited. QuickSee and Shin‐Nippon autorefractors were used to measure refractive status at distance (4 m) and near (50, 33 and 25 cm). Accommodative response was calculated as the difference between distance autorefraction and the value obtained for each accommodative demand. Individual accommodative response slopes were calculated from linear regression for response against demand and averaged to calculate mean slopes for each autorefractor. Inter‐instrument agreement between the QuickSee and Shin‐Nippon was assessed using the Bland–Altman method and 95% limits of agreement. Differences in measured accommodative responses between devices across accommodative demand were assessed by repeated measures ANOVA.

**Results:**

Forty‐nine children with a median (IQR) spherical equivalent refraction of +0.38 (0.50 D) (range −0.63 to +1.50 D) participated. Median (IQR) accommodative responses for the 2, 3 and 4 D demands were 1.63 D (0.69), 2.50 D (0.75) and 3.13 D (0.63), respectively, for QuickSee, and 1.63 D (0.69), 2.50 D (0.56) and 3.25 D (0.63) for the Shin‐Nippon. Mean ± SD slopes for accommodative response were 0.75 ± 0.22 and 0.83 ± 0.18 for QuickSee and Shin‐Nippon, respectively (*p =* 0.09). Pearson's correlation showed no significant proportional bias for measures of accommodative response with either autorefractor (*p* ≥ 0.19). The mean difference in accommodative response measured by the QuickSee and Shin‐Nippon did not differ significantly with accommodative demand (*F* = 1.70, *p* = 0.19).

**Conclusion:**

The agreement observed here demonstrates that the QuickSee shows promise in measuring accommodative response over a range of demands (2, 3 and 4 D) in a paediatric population without significant ametropia. Further research is required to determine if these findings translate over a wider range of refractive errors, and to evaluate the value of QuickSee accommodative response measures in vision screening.

**Key points**

- This study evaluated the QuickSee autorefractor for measurement of accommodative response in a cohort of children aged 5–7 years without significant ametropia.
- The QuickSee autorefractor demonstrated good agreement with the gold‐standard Shin‐Nippon device for measurement of the accommodative response over a range of demands (2, 3 and 4 D).
- These findings support the use of the QuickSee autorefractor for measurement of the accommodative response; however, further work is required to evaluate if these findings translate across a larger range of refractive errors.

## INTRODUCTION

Uncorrected refractive error continues to be the leading cause of visual impairment worldwide, imposing on public health, education and an individual's ability to work.^1^ Therefore, there is a need for sensitive vision screening tests which can detect uncorrected refractive error accurately during childhood to allow for early intervention.

With the majority of research relating to childhood refractive error focussed on the rising prevalence of myopia, hypermetropia (hyperopia) is often overlooked.^2^, ^3^ In the United Kingdom, the Northern Ireland Childhood Errors of Refraction study reported levels of hyperopia (defined as spherical equivalent refraction ≥+2.00 DS) of 26% (95% CI 20–33%) and 14.7% (95% CI 9.9–19.4%) in children aged 6–7 and 12–13 years, respectively.^4^ The prevalence of hyperopia varies with geographical location, with estimated pool prevalence in children ranging from 2.2% in Southeast Asia to 14.3% in the Americas.^4^, ^5^, ^6^

In the United Kingdom, the National Screening Committee recommends that standardised vision screening should be offered to all children aged 4–5 years.^7^ This screening is predominantly focused on the detection of amblyopia.^8^ A lack of both evidence and agreement exists regarding the best battery of tests, which should be used to detect a broad range of visual conditions at an affordable cost.^8^, ^9^, ^10^ Therefore, recommendations currently suggest that children should be screened using distance visual acuity only. The United Kingdom is not alone in this practice.^10^

While the sensitivity of distance visual acuity to detect amblyopia and myopic refractive errors is excellent, this measurement has low sensitivity to detect uncorrected hyperopia.^11^, ^12^ In a sample of 392 children aged 6–7 years, reduced uncorrected distance visual acuity (poorer than 0.20 logMAR) detected hyperopia >+3.50 D with sensitivity of 54% and specificity of 91%.^11^ This poor sensitivity arises due to the presence of active accommodation in childhood, whereby the eye adjusts its dioptric power to change focus and maintain clear vision. Through exertion of accommodation, young hyperopic individuals can often overcome their visual symptoms of blur and maintain good distance visual acuity.

Given the negative educational impact of uncorrected hyperopia and the potential benefits of refractive correction,^13^, ^14^, ^15^, ^16^, ^17^, ^18^, ^19^ identification of more sensitive screening tests for uncorrected hyperopia which are objective, cost‐effective, portable and require minimal training to operate would be valuable for both paediatric vision screening and vision science research.

Recent work has suggested that measurement of accommodative function alongside distance visual acuity in vision screening may have potential to increase the signal for detecting hyperopia. Underaccommodation to a near target is often referred to as ‘accommodative lag’. The Vision in Pre‐schoolers‐Hyperopia in Pre‐schoolers (VIP‐HIP) study demonstrated a consistent trend of larger lags of accommodation with increasing hyperopia in children aged 4–5 years.^20^ Likewise, Candy et al.^21^ reported greater and more variable lags of accommodation in children aged 3.7–90 months with >+4.00 D of hyperopia compared to those of the same age with ≤+4.00 D of refractive error. Furthermore, a lag criterion of 1.3 D detected hyperopia >5 D in any meridian, amblyopia and/or strabismus with sensitivity of 83.3% and a specificity of 96.5%. Other aspects of visual function such as visual acuity and stereopsis may also be reduced in children with uncorrected hyperopia.^12^, ^15^, ^22^

Accommodative function may be measured using subjective or objective methods; however, the former requires the subjective determination of blur by the subject, as with measurement of the amplitude of accommodation, and therefore risks overestimating the true value due to depth of field effects.^23^, ^24^ The timing of this test as well as variation in the patient's interpretation and clarity of instructions may also lead to measurement inaccuracies. Dynamic retinoscopy (Nott or Monocular Estimated Methods) is an alternative, objective technique to assess the accuracy of accommodative response to a near target. It is particularly useful when examining younger children or subjects with limited cooperation. However, it requires a highly skilled examiner to interpret the retinoscopy reflex appropriately and is therefore not suitable for use in mass vision screening programmes.^25^

An objective method that, to date, has been chiefly employed in vision science research, is the use of an open‐field autorefractor to measure the refractive status of the eye when viewing distance and near targets. Accommodative response is calculated as the difference between refractive states at distance and near.^26^ The gold‐standard autorefractor for this measurement is the Shin‐Nippon (Shin‐Nippon NVision‐K 5001; Rexxam Co. Ltd., rexxam.co.jp), an open‐field autorefractor which has demonstrated high accuracy and reliability for measures of refractive error and accommodative responses among both adults and children.^27^, ^28^, ^29^ However, the Shin‐Nippon is relatively expensive and bulky, requires mains electrical power and is table‐mounted, making it unsuitable for transport during research field work and school vision screenings.

The present study aims to investigate the reliability of a novel autorefractor, the QuickSee (QuickSee; PlenOptika Inc., plenoptika.com), for measurements of accommodative response in children. The QuickSee is a commercially available device based on Shack‐Hartmann wavefront aberrometry technology. Like the Shin‐Nippon, the QuickSee has an open‐field design. It is handheld, battery powered, requires minimal training to operate and is less expensive than other handheld autorefractors on the market. These features would make the QuickSee an ideal instrument for use in a school vision screening setting.^8^, ^9^, ^10^, ^30^, ^31^

When previously evaluated in paediatric populations, the QuickSee autorefractor has shown good agreement with both cycloplegic and non‐cycloplegic subjective refraction and has a high testability of 94.4% among young children aged 4–12 years.^32^, ^33^ The QuickSee also yields favourable results when compared with retinoscopy and other handheld autorefractors for measures of refractive error among adult populations.^34^, ^35^, ^36^ However, there is a paucity of research evaluating the potential of the QuickSee to measure the accommodative response.^37^

In order to explore further the potential for accommodative measurements to aid in the detection of hyperopia within a vision screening or research setting, one must first identify and evaluate low‐cost, portable devices capable of measuring objective accommodative responses within a paediatric population. The present study aimed to investigate the potential of the QuickSee to measure the accommodative response in a healthy paediatric population, evaluating the QuickSee autorefractor for measures of accommodative response in a cohort of children without significant ametropia and comparing these measures to those obtained using the gold‐standard Shin‐Nippon device.

## METHODS

### Participants

Participants were children aged 5–7 years recruited from a local primary school. Study information was sent home to parents seeking healthy children with good eyesight. Informed written parental consent and written participant assent were obtained prior to commencing the study procedures. The study was approved by the Ulster University Research Ethics Committee and adhered to the tenets of the Declaration of Helsinki.

Sample size was determined using a power calculation for ‘agreement between two methods’ studies^38^ as below:

Desired confidence interval of limits of agreement = $1.96\sqrt{\frac{3s^{2}}{n}}$.

*s* = standard deviation of mean difference between methods.

*n* = sample size.

For a desired confidence interval < 0.25 D (commonly used step size for subjective refraction) and a standard deviation of 0.51 D (for mean difference between accommodative response measured with dynamic retinoscopy and Shin‐Nippon SRW‐5000 Autorefractor^25^), a required sample size of 48 participants was calculated.

### Procedures

Data collection took place over one study visit on the school campus, during the school day. Children's age was elicited using the consent form completed by the parents. A brief medical history was taken to elicit whether the children had any systemic or ocular conditions, were taking medication or had a diagnosis of a learning difficulty/disability or developmental disorder. Exclusion criteria included a history of systemic or ocular disease or medications that may impact on the child's accommodative ability, for example antihistamine or diabetes mellitus medication. Children with a learning difficulty/disability or developmental disorder, which may impact on their sustained attention were also excluded. All techniques involved were performed by the same examiners (RL and TDU), both experienced optometrists.

Consented children attended for the following tests. All measurements were taken with spectacles, if worn:

- Presenting distance and near monocular visual acuity (VA), measured using a linear crowded logMAR test chart (Bailie‐Lovie or LEA symbols at 3 m for distance; Sloan or LEA symbols at 40 cm for near).
- Distance and near cover test at 3 m and 33 cm, respectively, to screen for the presence of heterotropia.

Conventional distance autorefraction was performed using the Shin‐Nippon instrument to measure refractive error in both eyes while the participant viewed a distance target (Maltese cross) at 4 m.

Subsequently, participants were excluded if the habitual distance or near VA was worse than 0.20 logMAR in the right eye, or if there was strabismus, myopia (spherical equivalent refraction (SER) < −0.75 D) and/or astigmatism >1.50 D. If significant refractive error or reduced VA not corrected with spectacles was detected, or in any other case deemed necessary by the study investigators who were registered optometrists, then a letter of information was sent to the parents to advise them to take the child to their local optometrist for clinical care.

### Experimental set up for accommodative response measures

Distance and near autorefraction measures used to calculate accommodative responses were obtained through spectacles lenses, if worn, using the Shin‐Nippon (Shin‐Nippon NVision‐K 5001; Rexxam Co. Ltd., rexxam.co.jp) and QuickSee (QuickSee; PlenOptika Inc., plenoptika.com) autorefractors. Data from paired eyes are highly correlated,^38^ hence QuickSee autorefraction was performed for the right eye only, unless participants exhibited >1.00 D of anisometropia by Shin‐Nippon findings, in which case measurements were obtained from the least hyperopic eye. The step size for both instruments was set to ±0.25 D.

During near autorefraction measurements, the participant fixated on a near target held at consecutively closer working distances (50, 33 and 25 cm, equating to approximately 2, 3 and 4 D accommodative demands, respectively). Accommodative demand is approximate as children without glasses may have had small amounts of uncorrected refractive error. The order in which the QuickSee and Shin‐Nippon measurements were performed was reversed for sequential participants.

**FIGURE 1:**
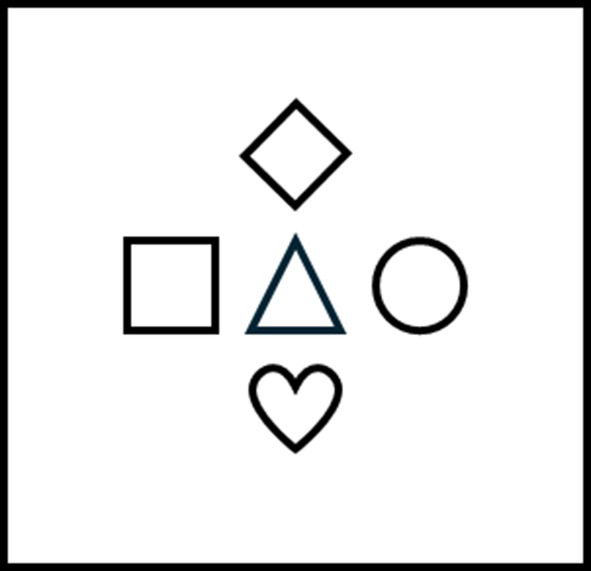
Example of the age‐appropriate accommodative target, which was displayed in front of the Shin‐Nippon and QuickSee autorefractors. The position of the symbols (square, triangle, diamond, circle and heart) within the cross was refreshed every 2 s.

The near target was presented on a back‐illuminated organic light emitting diode (OLED) display (Iphone11, Apple Inc., apple.com) and consisted of age‐appropriate symbols (each 3 mm × 3 mm) designed to be salient accommodative targets. At 25 cm, each symbol corresponded to 0.30 logMAR. Symbol size was not adjusted for working distance. To ensure that attention was sustained during the testing procedures, the symbols were refreshed every 2 s and participants named the symbol in the centre of each target. An example of the age‐appropriate accommodative target utilised is shown in Figure 1.

The sphero‐cylindrical representative values given by the autorefractors were used to calculate the SER using the following equation: SER = Sphere + Cyl/2. The accommodative response (in dioptres) for each test distance was calculated as follows: Accommodative response = distance SER − near SER at 2, 3 or 4 D demand.

### Statistical analysis

Data were transferred from hard copy data collection forms to electronic format using Excel (Microsoft Excel for Mac, version 16.82, Microsoft.com), cleaned and then analysed using IBM SPSS statistical software (version 29, ibm.com). Distributions of the data were assessed using the Shapiro–Wilk test.

For each participant, linear regression was used to calculate the slope of the accommodative response against the demand and derive a single measure of accommodative performance for each instrument. The mean QuickSee and Shin‐Nippon regression lines (slopes) for all participants were compared using a paired samples *t*‐test. This study focused on accommodation to near stimuli; therefore, the 0 D point (which relates more closely to baseline tonic accommodation) was not included in the calculation of the accommodative slope.

Mean differences, standard deviations (SD) and 95% limits of agreement (LOA) were calculated for all measures of accommodative response, and Bland and Altman plots were used to illustrate the agreement between the QuickSee and Shin‐Nippon autorefractors. Bland and Altman plots present the difference between the two measurements along the Y‐axis and the mean of the measurements along the X‐axis. 95% LOA were calculated using the following equation: 95% LOA = mean difference ± (1.96 × SD_diff_).^39^ Reference lines were added to the Y‐axis to represent the mean difference between two measurements, upper and lower 95% LOA and their 95% confidence intervals. Pearson's correlations were used to determine the presence of proportional bias. A *p*‐value <0.05 was considered statistically significant. Differences in the accommodative response between the QuickSee and Shin‐Nippon autorefractors across accommodative demand were assessed by repeated measures ANOVA.

## RESULTS

Screening was carried out on 64 children, of whom 49 met the inclusion criteria to participate in the study and completed all measurements. Eligible children had a mean age of 6.35 ± 0.53 years (range 5.30–7.26 years) and 47% were male.

The median ± interquartile range (IQR) distance VA was 0.04 ± 0.14 and 0.02 ± 0.16 logMAR for the right and left eyes, respectively. Median ± IQR near VA was 0.00 ± 0.13 and 0.00 ± 0.12 logMAR for the right and left eyes, respectively. Distance and near VA were positively correlated in both eyes, reaching statistical significance in the left eye (rho = 0.32, *p* = 0.03), but not in the right eye (rho = 0.17, *p* = 0.24).

Conventional (i.e., uncorrected) distance autorefraction using the Shin‐Nippon device showed a median ± IQR SER of +0.38 ± 0.50 D (range −0.63 to +1.50 D) and +0.50 ± 0.50 D (range −0.25 to +3.25 D) for the right and left eyes, respectively. Median ± IQR astigmatism was −0.50 ± 0.25 D (range −1.25 to 0 D) and −0.50 ± 0.50 D (range −1.50 to 0 D) for right and left eyes, respectively. Median ± IQR anisometropia (the difference between the spheres recorded for the right and left eyes) was 0.50 D (range 0.0–2.50 D).

No participants exhibited anisometropia >1.00 D by Shin‐Nippon autorefraction where the least hyperopic eye was the left eye; therefore, QuickSee distance autorefraction and accommodative measurements were obtained from the right eye only for all participants. Two eligible children were observed to be wearing spectacles habitually on the day of data collection, and therefore, distance autorefraction measures were obtained both uncorrected (Shin‐Nippon autorefractor only) and through their spectacle lenses (both Shin‐Nippon and QuickSee autorefractors). The median ± IQR SER distance autorefraction (through spectacles, if worn) was 0.00 ± 0.38 D with the QuickSee and +0.38 ± 0.50 D with the Shin‐Nippon autorefractor (Wilcoxon signed‐rank test, *p* < 0.001).

Table 1 presents the median ± IQR accommodative response for the Shin‐Nippon and QuickSee autorefractors for 2, 3, and 4 D accommodative demands. The mean difference in accommodative response measured with the two devices was not significant for accommodative demands of 2, 3 and 4 D (repeated measures ANOVA, *F*(2, 96) = 1.70, *p* = 0.19).

**TABLE 1:** Median ± IQR accommodative response measured by the QuickSee and Shin‐Nippon autorefractors for accommodative demands of 2, 3 and 4 D in children aged 5–7 years.

Dioptric demand (D)	Median accommodative response (IQR) (D)	
	QuickSee	Shin‐Nippon
2	1.63 (1.31–2.00)	1.63 (1.31–2.00)
3	2.50 (2.00–2.75)	2.50 (2.19–2.75)
4	3.13 (2.81–3.44)	3.25 (3.00–3.63)

Abbreviations: D, dioptres; IQR, interquartile range.

**FIGURE 2:**
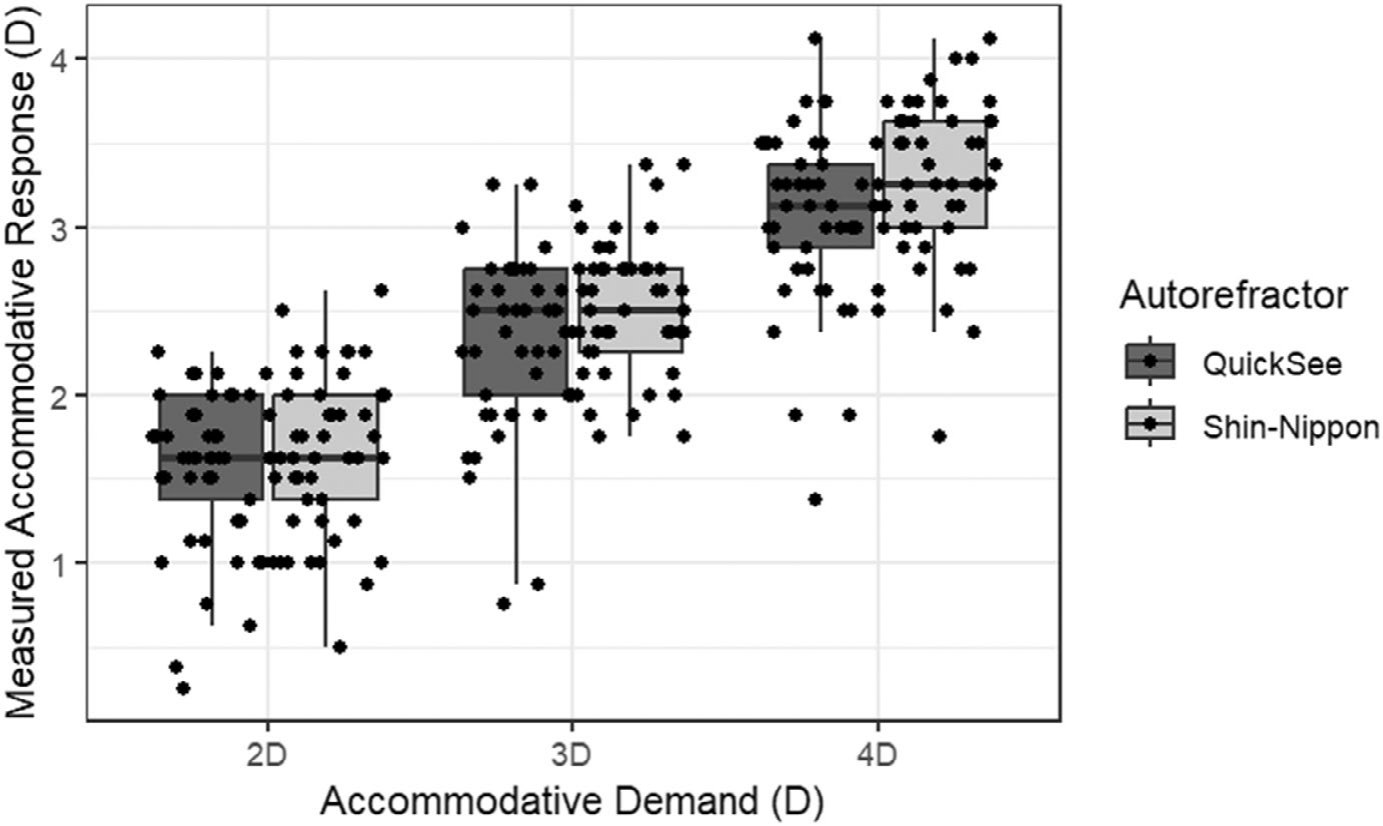
Boxplots illustrating the accommodative response measured at demands of 2, 3 and 4 D using the QuickSee (dark grey) and Shin‐Nippon (light grey) autorefractors in children aged 5–7 years. Upper and lower bounds of boxes represent the 25th and 75th percentiles of the accommodative response, while the line inside the box represents the median (50th percentile). Upper and lower limits (Q3 + 1.5 × IQR and Q1–1.5 × IQR, respectively) represent the minimum and maximum accommodative response when outliers are excluded. Individual data points are represented by black dots.

Figure 2 shows boxplots depicting the median and interquartile range of the accommodative response measured at each accommodative demand for the two instruments.

**FIGURE 3:**
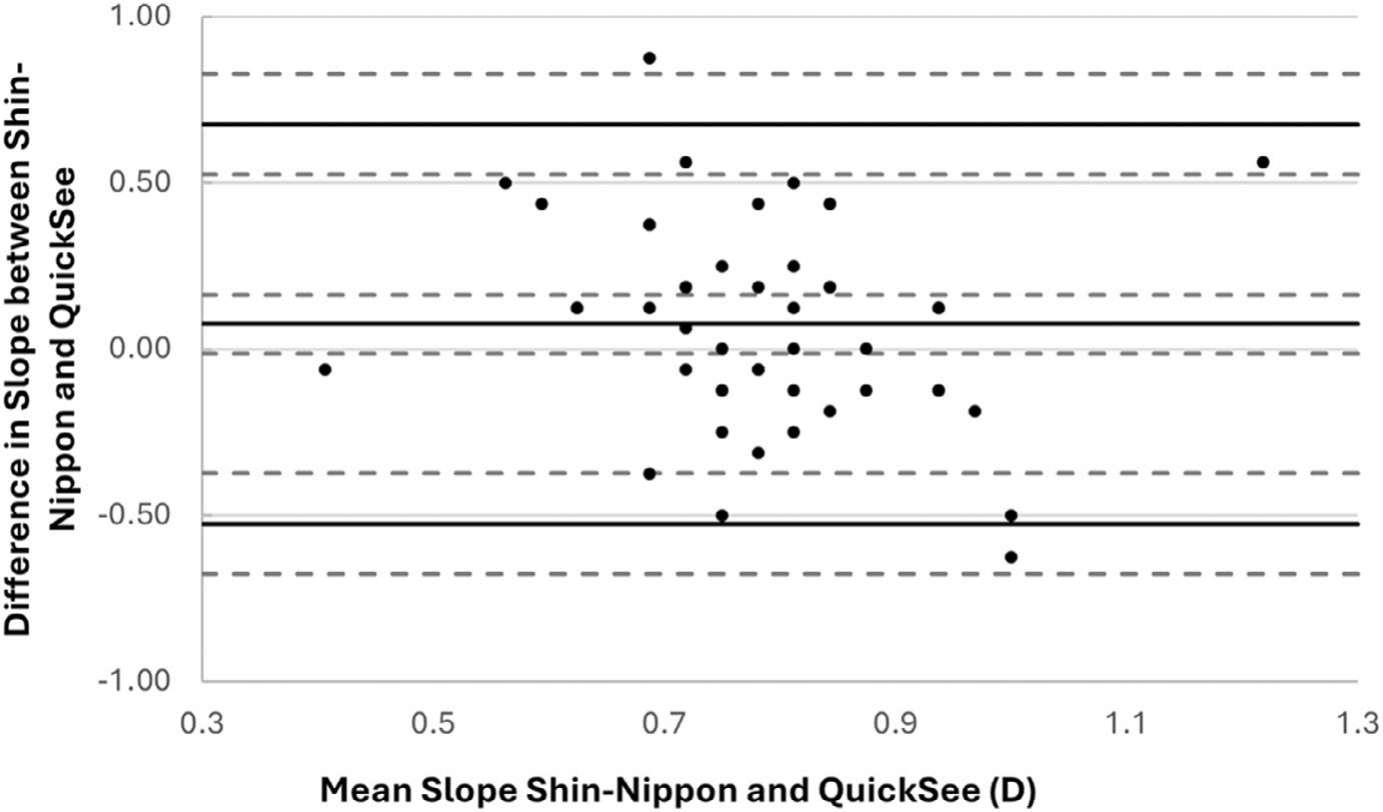
Bland and Altman plot showing the difference in the slope of the accommodative response, with respect to the accommodative demand, obtained by the QuickSee and Shin‐Nippon autorefractors. Reference lines represent the mean difference, upper and lower 95% LOA (all solid lines) and their 95% confidence intervals (dashed lines). The mean difference was calculated by subtracting the slope obtained with the QuickSee instrument from that determined using the Shin‐Nippon autorefractor.

The accommodative slope refers to the rate of change of the accommodative response with increasing accommodative demand. Overall mean (±SD) accommodative slopes for the QuickSee and Shin‐Nippon were 0.75 ± 0.22 and 0.83 ± 0.18, respectively, with a paired samples t‐test showing that this difference was not significant (*p =* 0.09). Figure 3 shows a Bland Altman plot comparing the differences in the slope.

**FIGURE 4:**
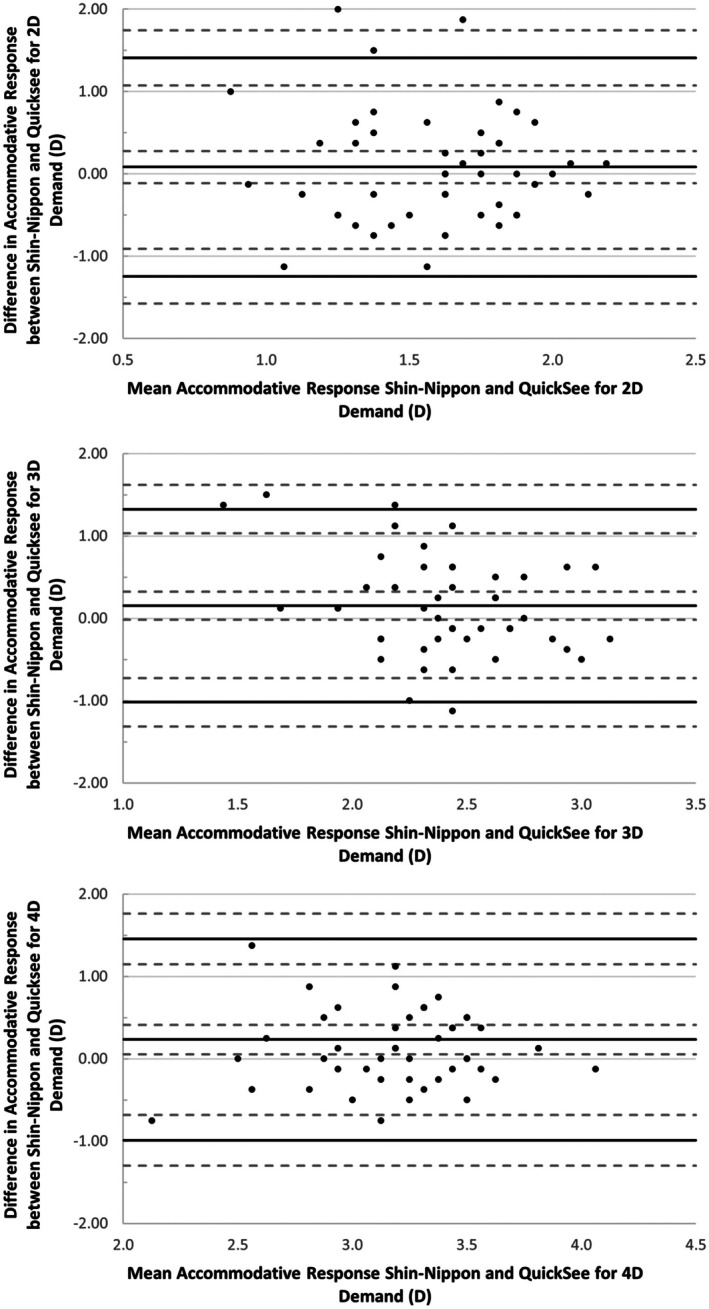
Bland Altman plots showing the difference between the accommodative response measurements for 2, 3 and 4 D demands obtained with the QuickSee and Shin‐Nippon autorefractors. Reference lines represent the mean difference between the two measurements, upper and lower 95% limits of agreement (LOA, all solid lines) and their 95% confidence intervals (dashed lines). The mean difference was calculated by subtracting the accommodative response measured with the QuickSee instrument from that obtained using the Shin‐Nippon autorefractor.

The mean difference, SD and 95% LOA for the accommodative response measured by the QuickSee and Shin‐Nippon instruments are presented in Table 2. Bland and Altman plots showing the difference in individual accommodative response at 2, 3 and 4 D demands as measured by the QuickSee and Shin‐Nippon autorefractors are shown in Figure 4. Pearson's correlation showed no significant proportional bias for these slopes, or for the accommodative responses at any of the dioptric demands tested here using the QuickSee and Shin‐Nippon autorefractors (all *p* ≥ 0.19).

**TABLE 2:** Agreement in accommodative response measurements (SER) taken by the QuickSee and Shin‐Nippon autorefractors at 2, 3 and 4 D dioptric demands.

Dioptric demand (D)	Mean difference (SD) (D)	Upper 95% LOA (95% CI)	Lower 95% LOA (95% CI)
2	0.08 (0.68)	1.41 (1.07–1.74)	−1.24 (−1.58 to −0.91)
3	0.15 (0.60)	1.32 (1.03–1.62)	−1.02 (−1.32 to −0.72)
4	0.23 (0.62)	1.46 (1.15–1.76)	−0.99 (−1.30 to −0.68)

*Note*: Mean difference was calculated by subtracting accommodative response measured by QuickSee from Shin‐Nippon.

Abbreviations: CI, confidence intervals; D, dioptres; LOA, limits of agreement; SD, standard deviation; SER, spherical equivalent refraction.

## DISCUSSION

The present study evaluated the novel QuickSee autorefractor for measurements of accommodative response in a sample of healthy children aged 5–7 years without significant ametropia, and compared its outputs with those obtained using the currently accepted gold‐standard Shin‐Nippon autorefractor. The QuickSee demonstrated good agreement with the Shin‐Nippon for measures of accommodative response to 2, 3 and 4 D demands, indicated by small mean differences of 0.08 ± 0.68, 0.15 ± 0.60 and 0.23 ± 0.62 D, respectively, with satisfactory 95% LOA (2 D demand, −1.24 to +1.41 D; 3 D demand, −1.02 to +1.32 D; 4 D demand, −0.99 to +1.46 D). The differences in measured accommodative responses between devices did not differ significantly across accommodative demands (*p* = 0.19).

Although not clinically significant, the small differences found between the accommodative response measurements obtained by the QuickSee and Shin‐Nippon may be explained by the different technology used in these instruments. The Shin‐Nippon autorefractor uses infrared light technology to determine the refractive state of the eye while the subject views an externally placed target, and the examiner is required to align the autorefractor centrally with the eye and instruct the patient to gaze at the desired target. The Shin‐Nippon uses a ring target of infrared light (850 nm) projected onto the retina. The reflected beam containing error information from the eye travels along the same path back toward a focusing lens on a motorised track, which places the ring approximately in focus. Finally, digital analysis of the ring image in multiple meridians calculates the refractive error of the eye.^40^ In contrast, the QuickSee is based on Shack‐Hartmann wavefront aberrometry, which offers detailed measurements of the movement of the light wavefront. The light passes through the pupil and is reflected back from the retina. The wavefront of the reflected beam is recorded and compared to the reference wavefront. Distortions that occur as light travels through the eye, that is aberrations, represent specific refractive errors.^36^

Unlike the Shin‐Nippon instrument, in which infrared measurement rings have a combined diameter of 2.32 mm, the wavefront aberrometry measurement technique used in the QuickSee autorefractor assesses the entire natural pupil area (acceptable pupil sizes range from 2 to 8 mm). While the device is designed to average wavefront data across the pupil, the residual effect of spherical aberration may influence accommodative measurements, especially in larger pupils. The shift to more negative spherical aberration with accommodation may also have contributed to the small differences observed in measured accommodative responses between the Shin‐Nippon and QuickSee devices.^41^, ^42^, ^43^, ^44^

Previous research has established the QuickSee autorefractor to be a robust method of quantifying refractive error in children, demonstrating good agreement with both cycloplegic and non‐cycloplegic subjective refraction.^32^, ^33^ The present study is the first to investigate the capacity of the QuickSee to measure the accommodative response in children. However, the present findings are consistent with work by Tran et al. comparing accommodative responses measured by the same devices for demands of 1, 2 and 3 D among adults aged 21–35 years (*n* = 30).^37^

A strength of the current study is the inclusion of a cohort with a good level of vision without significant ametropia. Furthermore, participants were all children within a tight age range of 5–7 years. Although the current study evaluated the accommodative response over a range of accommodative demands (approximately 2, 3 and 4 D), it is possible that young children, especially those with uncorrected hyperopia, may exert higher accommodative efforts in everyday visual tasks than those evaluated under test conditions.

Given that larger lags of accommodation have been found in individuals with uncorrected hyperopia,^21^ measuring accommodative function alongside distance visual acuity in school vision screenings may increase the ability to detect uncorrected childhood hyperopia. This refractive error is commonly overlooked, but has been shown to have a negative impact on near visual function, early literacy development,^14^, ^45^ reading speed^16^ and academic achievement.^17^, ^18^, ^19^, ^46^, ^47^ Two interventional studies have reported hyperopic spectacle correction to improve reading speed significantly,^16^, ^48^ with some low‐moderate hyperopic children (cycloplegic retinoscopy ≥+1.00 and <+5.00 D) aged 5–10 years demonstrating more accurate and stable accommodative responses during sustained reading following refractive correction.^48^ Although further research is required to evaluate the impact of providing a hyperopic correction on academic performance,^13^ these findings suggest that screening for clinically significant levels of hyperopia may be worthwhile in countries with significant prevalence of the condition.

While there is some precedent for the inclusion of accommodative measurements within vision screening research, accommodative measures have not yet been widely adopted within school vision screening programmes. The New York State Optometric Association (NYSOA) battery, developed in 1983, incorporates visual efficiency tests such as amplitude of accommodation. In school‐aged children (kindergarten through grade 5), an amplitude of accommodation < (15 D – [0.25 × age]) warrants referral.^49^ More recently, measurement of accommodative lag was included in the VIP‐HIP study.^20^ Completing such a battery of tests is time consuming, and measurement of the amplitude of accommodation relies on subjective responses from the children. In contrast, instrument‐based screening tests are quick, require minimal cooperation from the child and can facilitate measurement in preverbal, preliterate or developmentally delayed children. This study highlights the potential for the QuickSee portable autorefractor to overcome these obstacles and serve as a valuable instrument for field‐based measurement of accommodative response as part of vision screening.

## CONCLUSIONS

To date, the QuickSee autorefractor has been established to be comparable with retinoscopy, subjective refraction and commonly used table‐mounted autorefractors.^33^, ^34^, ^36^, ^50^ The present study demonstrates that the QuickSee autorefractor is a valid instrument for measuring accommodative responses over a range of demands (2, 3 and 4 D) among a healthy paediatric population without significant ametropia.

Measurement of distance visual acuity is the accepted screening method for detecting amblyopia and refractive error in children in many countries. However, this method has poor sensitivity for detecting hyperopic refractive errors.^11^ Testing the potential for objective accommodative measures to support the screening and detection of hyperopia will require a device which is objective, cost‐effective, portable and requires minimal training to operate. If further research confirms the agreement with gold‐standard measurements of accommodative response demonstrated in this study to translate over a wider range of refractive errors, then the QuickSee autorefractor is likely to fulfil these requirements.

## AUTHOR CONTRIBUTIONS

**Jane M. Fulton:** Formal analysis (equal); visualization (equal); writing – original draft (equal); writing – review and editing (equal). **Rebecca E. Leighton:** Conceptualization (equal); formal analysis (equal); investigation (equal); methodology (equal); project administration (equal); writing – original draft (equal); writing – review and editing (equal). **Tuyishime Didier Fidele Uwacu:** Investigation (equal); methodology (equal); writing – review and editing (equal). **Bruce Moore:** Conceptualization (equal); methodology (equal); writing – review and editing (equal). **Julie‐Anne Little:** Conceptualization (equal); methodology (equal); project administration (equal); supervision (equal); writing – review and editing (equal).

## FUNDING INFORMATION

The study was performed with the in‐kind support of equipment and resources from the Centre for Optometry and Vision Science (School of Biomedical Sciences, Ulster University) for the research associate and team; no additional funding was provided.

## CONFLICT OF INTEREST STATEMENT

The authors declare no conflict of interest.
